# Organ failure and tight glycemic control in the SPRINT study

**DOI:** 10.1186/cc9224

**Published:** 2010-08-12

**Authors:** J Geoffrey Chase, Christopher G Pretty, Leesa Pfeifer, Geoffrey M Shaw, Jean-Charles Preiser, Aaron J Le Compte, Jessica Lin, Darren Hewett, Katherine T Moorhead, Thomas Desaive

**Affiliations:** 1 Department of Mechanical Engineering, Centre for Bio-Engineering, University of Canterbury, Christchurch, Private Bag 4800, 8054, New Zealand; 2 University of Otago Christchurch, School of Medicine, Christchurch, 8054, New Zealand; 3 Department of Intensive Care, Christchurch Hospital, Christchurch, 8054, New Zealand; 4 Department of Intensive Care, Centre Hospitalier Universitaire de Liege (CHU de Liege), B4000 Liege, Liege, Belgium; 5 Cardiovascular Research Centre, Institute de Physique, Universite de Liege, Institute of Physics, Allée du 6 Août, 17 (Bât B5), B4000 Liege, Liege, Belgium

## Abstract

**Introduction:**

Intensive care unit mortality is strongly associated with organ failure rate and severity. The sequential organ failure assessment (SOFA) score is used to evaluate the impact of a successful tight glycemic control (TGC) intervention (SPRINT) on organ failure, morbidity, and thus mortality.

**Methods:**

A retrospective analysis of 371 patients (3,356 days) on SPRINT (August 2005 - April 2007) and 413 retrospective patients (3,211 days) from two years prior, matched by Acute Physiology and Chronic Health Evaluation (APACHE) III. SOFA is calculated daily for each patient. The effect of the SPRINT TGC intervention is assessed by comparing the percentage of patients with SOFA ≤5 each day and its trends over time and cohort/group. Organ-failure free days (all SOFA components ≤2) and number of organ failures (SOFA components >2) are also compared. Cumulative time in 4.0 to 7.0 mmol/L band (cTIB) was evaluated daily to link tightness and consistency of TGC (cTIB ≥0.5) to SOFA ≤5 using conditional and joint probabilities.

**Results:**

Admission and maximum SOFA scores were similar (*P *= 0.20; *P *= 0.76), with similar time to maximum (median: one day; IQR: [1,3] days; *P *= 0.99). Median length of stay was similar (4.1 days SPRINT and 3.8 days Pre-SPRINT; *P *= 0.94). The percentage of patients with SOFA ≤5 is different over the first 14 days (*P *= 0.016), rising to approximately 75% for Pre-SPRINT and approximately 85% for SPRINT, with clear separation after two days. Organ-failure-free days were different (SPRINT = 41.6%; Pre-SPRINT = 36.5%; *P *< 0.0001) as were the percent of total possible organ failures (SPRINT = 16.0%; Pre-SPRINT = 19.0%; *P *< 0.0001). By Day 3 over 90% of SPRINT patients had cTIB ≥0.5 (37% Pre-SPRINT) reaching 100% by Day 7 (50% Pre-SPRINT). Conditional and joint probabilities indicate tighter, more consistent TGC under SPRINT (cTIB ≥0.5) increased the likelihood SOFA ≤5.

**Conclusions:**

SPRINT TGC resolved organ failure faster, and for more patients, from similar admission and maximum SOFA scores, than conventional control. These reductions mirror the reduced mortality with SPRINT. The cTIB ≥0.5 metric provides a first benchmark linking TGC quality to organ failure. These results support other physiological and clinical results indicating the role tight, consistent TGC can play in reducing organ failure, morbidity and mortality, and should be validated on data from randomised trials.

## Introduction

After the first two to three days of patient stay, mortality in the intensive care unit (ICU) and in-hospital are strongly associated with, and/or attributable to, organ failure and sepsis [1-3]. In particular, a lack of organ failure resolution over a patient's stay is associated with increased morbidity and mortality, as commonly measured by the sequential organ failure assessment (SOFA) score [4-6]. However, the specific mechanisms are not necessarily fully understood [7-10].

Blood glucose levels and their variability have also been associated with increased organ failure, morbidity and mortality, particularly in sepsis [11-14]. Hyperglycemia can have lasting impact at a cellular level, even in subsequent euglycemia, due to over production of superoxides [15], leading to further damage and complications. Hyperglycemia can also increase pro-inflammatory nitric oxide synthase activity, as part of the process that sees increased damage to the endothelium along with reduced microvascular circulation, and reduced organ perfusion, all of which can be potentially reversed with insulin [16,17]. Tight glycemic control (TGC) by intensive insulin therapy (IIT) has been successful at reducing mortality and/or organ failure in some prior studies [18-21]. There are also strong physiological links between reduced glycemic levels (and reduction in their variability), and improved immune response to infection [22-24] as well as reductions in organ failure [8]. It is particularly interesting to note that while mortality was reduced for patients with length of stay three days or longer, differences in Kaplan-Meier plots do not appear before 10 to 15 days for these studies. These results suggest that earlier resolution of organ failure and dysfunction, and the resulting reduced morbidity, is a leading cause of at least part of the improvement. Additionally, while some studies showed benefit from TGC, several others have not achieved similar results [25-27], and equally, did not necessarily achieve (where reported) the same affect in mitigating organ failure.

Hence, this study hypothesises that TGC can mitigate organ failure and severity more rapidly in the first days of intensive care as a platform for improved outcome.

To test this hypothesis, the data from the retrospective SPRINT glycemic control study [21] was revisited and SOFA scores calculated for all 784 patients considered in the study (371 on SPRINT and 413 retrospective matched patients) for each day of ICU stay. Organ failure was calculated daily using the SOFA score for each patient. This study analyses these SOFA score trajectories to determine if organ failure was mitigated more rapidly in our TGC cohort, indicating a potential reason for the improved mortality that appears later in the stay. Further analyses examine differences in survivors and non-survivors, as well as the number of organ failures and organ failure free days in each cohort.

## Materials and methods

### SPRINT protocol

SPRINT is a model-derived [28,29] TGC protocol developed from clinically validated computer models used for real-time control in the ICU [28-32]. Implemented at the Christchurch Hospital Department of Intensive Care in August 2005 [21], SPRINT has now been used on over 1,000 patients. In a clinical comparison to statistically matched retrospective cohorts, the SPRINT TGC intervention reduced hospital mortality for those patients staying three to five days in the ICU by 25 to 40% [21].

SPRINT is a unique TGC protocol that uses explicit control of both insulin and nutrition inputs. It thus controls carbohydrate intake in balance with the insulin given, which is the unique feature of this protocol compared to all others. Other TGC protocols leave carbohydrate intake to local standards and do not explicitly account for its intake, delivery route or total dose in trying to achieve glycemic control [33-35]. In particular, SPRINT modulates nutritional intake between 30 to 100% of a patient-specific goal feed rate based on ACCP/SCCM guidelines [36]. SPRINT also specifies only low-carbohydrate enteral nutrition formulas with 35 to 40% carbohydrate content, unless clinically specified otherwise in rare cases. SPRINT is thus primarily unique in explicitly specifying and using carbohydrate intake, within acceptable ranges [36-38] for TGC.

Equally importantly, SPRINT determines insulin and nutrition interventions based on (estimated) insulin sensitivity of the patient (1/insulin resistance), rather than strictly on blood glucose levels or/and changes. Hence, insulin and nutrition are given in balance, based on estimated response to the prior insulin and nutrition intervention, which is enabled by the protocols explicit knowledge of carbohydrate intake. The overall system thus matches the nutrition and exogenous insulin given to the body's patient-specific ability to utilise them, thus avoiding hyperglycemia. This approach is unique to SPRINT.

SPRINT also modulates interventions very slowly. Over 90% of interventions change insulin or nutrition rates by ± 1 U/hour and/or ± 10% (nutrition rate), or less. Further, large drops in blood glucose (>1.5 mmol/L with BG <7 mmo/L) trigger the shut off of insulin even though blood glucose is over the 6.0 mmol/L target. This relatively slow, very conservative approach is much less aggressive than almost all other protocols, minimising rapid changes in glycemia and thus hypoglycemia.

Finally, SPRINT measures more frequently than almost all other protocols. It specifies one or two hourly measurement and intervention intervals. This rate is also based on patient-specific insulin sensitivity. This feature is also unique compared to other protocols that typically utilise reaching a glycemic band or similar glycemic outcome to change measurement frequency. More specifically, it requires a patient to be *stable *which is defined as in the target band (4 to 6 mmol/L, target of 6 mmol/L) for three hours with higher than average insulin sensitivity (low insulin resistance), as assessed by receiving 3 U/hour or less of insulin and 60% or more goal nutrition rate. Hence, stability, and thus measurement frequency are a function of a patient's assessed insulin sensitivity as a broad marker of their level of wellness and potential variability. Equally, the protocol does not allow a four-hour measurement, as many others do, which ensures that glycemic control is not lost for patients who can demonstrate significant hourly metabolic variability [28,39,40].

As a result, SPRINT provided very tight control. In particular, it reported very high times in tight glycemic bands compared to other studies [41]. SPRINT also provided tight control more consistently across patients where the median blood glucose for the 25^th ^and 75^th ^percentile patients was separated by 1.1 mmol/L (1.9 mmol/L for the 5^th ^and 95^th ^percentiles). Overall, 97% of patients had 50% or more of their glucose values within a 4.0 to 7.0 mmol/L range. More importantly, while SPRINT gave more insulin it is the only reported study that reduced hypoglycemia (<2.2 mmol/L) in the tight control group (2% by patient a 50% reduction from Pre-SPRINT). It also had a lower carbohydrate load than Pre-SPRINT due the nutrition specified and its formulation. Finally, and perhaps most importantly, there was no statistical association within the SPRINT cohort between mortality and any glycemic metric (median, average, range, maximum), indicating that all patients received equal (tight) control, and that glycemia was no longer a significant factor in mortality, which was not the case for the retrospective cohort. Appendix A in Additional File 1 contains a more detailed description of SPRINT and specific, unique differences to other protocols and Table 1 has a selection of glycemic and intervention results from the study.

**Table 1:** Comparison of SPRINT and retrospective cohort baseline variables with glycemic control and intervention results

	Overall				
	Retrospective		SPRINT		*P*-value
Total patients	413		371		
Age (years)	64 (53 to 74)		65 (49 to 74)		0.53
% Male	59.1%		63.6%		0.19
APACHE II score	18 (15 to 23)		18 (15 to 24)		0.50
APACHE II risk of death	28.5% (14.2% to 49.7%)		25.7% (13.1% to 49.4%)		0.39
Diabetic history	71 (17.2%)		62 (16.7%)		0.86
LoS median, IQR (days)	3.8 (1.8 to 8.8)		4.1 (1.7 to 10.4)		0.94
Median BG (SD) (mmol/L)	7.2 (2.4)		6.0 (1.5)		<0.01
% BG in 4.4-6.1 mmol/L	30.0%		53.9%		<0.01
% BG in 4.0-7.0 mmol/L	49.6%		80.1%		<0.01
% BG < 2.2 mmol/L	0.2%		0.1%		<0.01
Mean insulin rate (U/hour)	1.2		2.8		<0.01
Mean nutrition (kcal/day)	1,599		1,283		<0.01
*APACHE III diagnosis*					
***Operative***	Num. patients	%	Num. patients	%	*P*-value
Cardiovascular	99	24%	76	20%	0.24
Respiratory	10	2%	9	2%	1.00
Gastrointestinal	53	13%	60	16%	0.18
Neurological	9	2%	7	2%	0.77
Trauma	8	2%	14	4%	0.12
Other (Renal, metabolic, orthopaedic)	4	1%	4	1%	0.88
***Non-operative***	Num. patients	%	Num. patients	%	*P*-value
Cardiovascular	41	10%	39	11%	0.79
Respiratory	77	19%	66	18%	0.76
Gastrointestinal	7	2%	10	3%	0.34
Neurological	33	8%	20	5%	0.15
Trauma	29	7%	32	9%	0.40
Sepsis	29	7%	17	5%	0.15
Other (Renal, metabolic, orthopaedic)	14	3%	17	5%	0.39

*P*-values computed using chi-squared and rank-sum tests where appropriate.

APACHE, Acute Physiology and Chronic Health Evaluation; BG, blood glucose (level); IQR, inter-quartile range; LoS, length of stay; SD, standard deviation.

Pre-SPRINT glycemic control consisted of a standard glucose sliding scale for which aggressiveness could be adjusted [28]. Measurement frequency was not specified, but was approximately every four hours across the cohort (Table 1). As seen in Table 1 it still provided relatively good glycemic control compared to some studies with an average value of 7.2 mmol/L. However, this may be misleading as results were highly variable across patients.

### Patient data

This study uses data from 371 patients treated on SPRINT (August 2005 to May 2007) and 413 patients from (January 2003 to August 2005) prior to SPRINT, as in the original study [21]. Patients were selected on a per-protocol basis, based on matching initial blood glucose levels criteria and being given insulin therapy. They were similar in age, sex, and APACHE III diagnosis, including a randomised analysis to ensure robustness. Table 1 shows the overall patient data for both groups, as well as a selection of glycemic and intervention results from the original study. Further details on the selection and analysis of these cohorts is in [21]. The Upper South Regional Ethics Committee New Zealand granted ethics approval for the audit, analysis and publication of this data.

### Organ failure assessment

Hospital records were examined for all patients and each day of their ICU stay. The total SOFA score [4,5,42] was calculated daily for each patient, taking the most abnormal value for each parameter in each 24 hr period of ICU stay. Where a data point was missing or not available for a component, a value was interpolated from surrounding data. In this study, the Glasgow Coma score reflecting central nervous system function was excluded due to its reported lack of robustness and unreliability [43-47], and it is thus not consistently recorded in Christchurch Hospital. Other studies have made a similar exclusion [48]. The remaining five SOFA component scores are each directly related to organ function or failure, and thus yield a maximum score of 20 (0 to 4 per metric). The parameters used assess renal, cardiovascular, liver, and respiratory function, and blood coagulation. A high SOFA score indicates a high level of organ dysfunction.

### Analysis and statistics

The primary goal is to retrospectively examine the impact of TGC in mitigating organ failure using the SOFA score. Thus, each cohort is evaluated in terms of the number of patients with total SOFA score less than 5 each day (scores of 0 to 1 per category on average). This value represents a low level of dysfunction. A literature survey shows that this cut-off value is well below mean or median reported values for admission or long-term average scores in several studies and is thus indicative of relatively well patients [5,27,42,49-52]. Further, some studies show that a value of 5 or less includes only the lowest scoring (least organ failure) 10 to 25% of patients, even when accounting for the missing central nervous system criterion in this study [5,52]. A further study used a cut-off of 7 as *relatively well *[50]. Hence, the cut-off value of 5 appears to represent a reasonable, potentially conservative, value to represent a relatively well patient with resolving organ failure, reduced morbidity and thus an increased likelihood of survival.

Data are also presented for each cohort in terms of total SOFA score and its variation over ICU days. Differences between survivors and non-survivors are also examined. The results for specific organ failure scores (SOFA component scores) are examined for any notable differences over time. Finally, organ failure free days (OFFD) are considered, defined as a day in which a patient has no SOFA component score greater than 2, where a SOFA component value of 3 or 4 indicates a failure of that particular organ system, as defined in other literature [3,5,48]. These latter results are thus also considered in terms of individual organ (component) failures (IOF). IOF counts the percentage of individual SOFA score components of 3 or 4 (failure) out of the maximum total possible organ failures (where Max = 5 components × total patient days). Thus, OFFD is a surrogate for the speed of resolution and/or prevention of organ failure in the cohort, while IOF is a complementary cohort-wide measure of total organ failures.

To delineate the particular patients affected and for which SOFA scores the greatest changes were seen over time, SOFA score distributions for each day are also presented. For conciseness and clarity, curves of mean SOFA score are shown over the first 14 days of ICU stay for each cohort. To illustrate any differences in the more critically ill patients with SOFA ≥5 or much higher, the mean plus one standard deviation line or 83^rd ^percentile is also shown. These figures thus indicate how TGC affects SOFA scores for more critically ill patients, rather than just the trend for the mean patient.

Where required, SOFA score data over time are compared using the non-parametric Wilcoxon sign-rank test. The non-parametric Wilcoxon rank-sum test is used to compare data distributions. The Fisher exact test is used to compare OFFD, IOF and SOFA mortality data. A statistical test value of *P *<0.05 is considered significant in all cases.

### Relating TGC and SOFA score

A patient-specific daily metric of control quality is needed to assess any link between effective TGC and SOFA outcome. For this analysis, cumulative Time in Band (cTIB) is defined as the percentage of time a patient's blood glucose has been in a specified band (cumulatively) up to that point in time. Good control was defined based on the 95^th ^percentile patient response in SPRINT as cTIB >0.50 (50%) within a 4.0 to 7.0 mmol/L band. Over 90% of SPRINT patients reach this level by Day 3, so this definition captures the SPRINT cohorts' glycemic control. Cumulative time in band was used as this study hypothesises that it is consistent, safe, and tight (to target and not variable) TGC under SPRINT that provided the foundation for improved organ failure.

Specifically, cTIB was determined each day for each patient, creating a data pair of (cTIB, SOFA) for each day. Thus, patients can be separated into good (cTIB ≥0.5) or poor (cTIB <0.5) control, and SOFA ≤5 or SOFA >5. To test the link between TGC and SOFA score we developed the conditional probability of SOFA ≤5 given good control (cTIB ≥0.5) or P(SOFA ≤5 | cTIB ≥0.5). These probabilities are out of 1.0, showing the association of good control with SOFA ≤5 for a given day. This value is plotted for each day and cohort along with the percent of total patients who achieve good control.

In addition, the joint probability of each group is also assessed. These joint probabilities cover all four combinations of cTIB AND SOFA score for each day, and thus sum to 1.0 across all four for a given day and cohort. These probabilities are defined in Equations 1 to 4:

(1) P(SOFA≤5∩cTIB≥0.5)=joint probability of SOFA≤5 and cTIB≥0.5

Where this joint probability is calculated for each day out of all patients in each cohort, showing those patients with low SOFA scores and good control.

(2) P(SOFA≤5∩cTIB<0.5)=joint probability of SOFA≤5 and cTIB<0.5

Where this joint probability is calculated for each day out of all patients in each cohort, showing those patients who had low SOFA scores despite poor control.

The joint probabilities in Equations 1 to 2 cover those patients who have low SOFA scores. Similarly for those who do not have low SOFA scores:

(3) P(SOFA>5∩cTIB≥0.5)=joint probability of SOFA>5 and cTIB≥0.5

Where this joint probability is calculated for each day out of all patients in each cohort, showing those patients with higher SOFA scores, despite good control.

(4) P(SOFA>5∩cTIB<0.5)=joint probability of SOFA>5 and cTIB<0.5

Where this joint probability is calculated for each day out of all patients in each cohort, showing those patients who had higher SOFA scores and poor control.

These four cases in Equations 1 to 4 define this paper's hypothesis of good control and reduced SOFA scores, but also show the other cases in which patients can appear. Thus, these probabilities define the gaps and differences between lines of SOFA ≤5 for each cohort on each day.

## Results

Glycemic control results for both cohorts were statistically different and are presented in [21] along with detailed cohort and mortality data. Table 2 presents admission and maximum SOFA scores, plus mortality data for the whole cohort across SOFA score. No statistically significant differences are seen due to low numbers, although raw mortality is lower in all but the very highest maximum SOFA score group. However, these are total cohort results, where the original study [19] only showed mortality differences for patients with ICU stay three days or longer.

**Table 2:** Day 1 and maximum total SOFA score for each cohort plus percent mortality and number of patients (died, lived) by maximum SOFA score range

	SPRINT	Pre-SPRINT	*P*-value
**Day 1 SOFA (Mean ± SD)**	5.6 ± 2.8	5.4 ± 3.0	0.20
**Maximum SOFA (Mean ± SD)**	6.8 ± 3.0	7.0 ± 3.2	0.76
**Day of Maximum SOFA score (Median (IQR))**	1 (1, 3)	1 (1, 3)	0.99
**Mortality (%) (#Died, #Lived) by maximum SOFA range**			
**0 to 4**	4.4% (4, 86)	5.2% (5, 92)	0.71
**5 to 9**	15.0% (32, 182)	15.3% (36, 199)	0.59
**10 to 14**	35.4% (22, 40)	40.8% (29,42)	0.79
**15 to 19**	75.0% (3, 1)	70.0% (7, 3)	0.79

IQR, inter-quartile range; SD, standard deviation; SOFA, Sequential Organ Failure Assessment.

**Figure 1:**
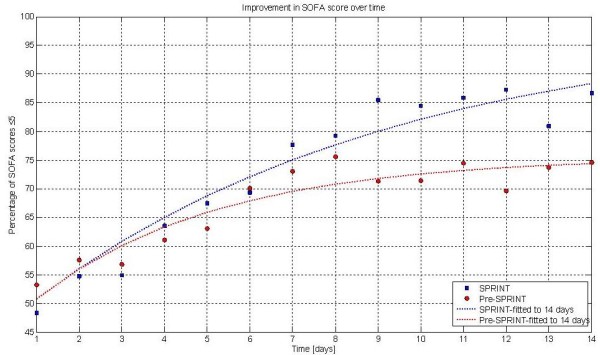
**Percentage of patients with SOFA ≤5 over each day (to 14 days)**. Exponential lines are fit to the data for clarity. Clinical data are significantly different (*P *≤0.001). Modifying the lines to fit over 21, 23, 25 and 28 days yields very similar curves and significant *P*-values (*P *< 0.04) in all these ranges.

**Figure 2:**
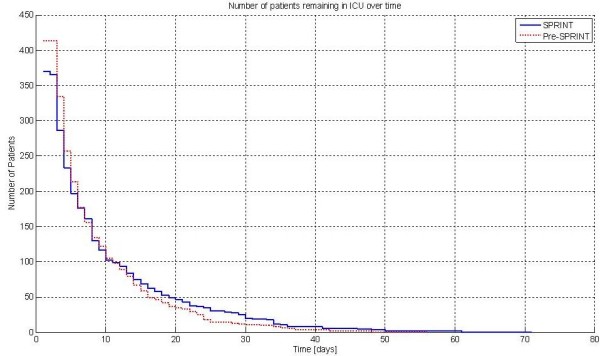
**Patients remaining by day**. At 14 days there are 67 Pre-SPRINT and 75 SPRINT patients remaining. The crossover in percentage of cohort remaining (not shown) is between Day 3 and Day 4.

Figure 1 presents the percentage of patients in each cohort with a total SOFA ≤5 for each of the first 14 days, showing organ failure resolution over time. The clinical data are significantly different over the first 14 days (*P *= 0.016). This data is fitted with an exponential curve for clarity. The clinical data are statistically different between cohorts (*P *< 0.04) for the data over the first 21, 23, 25 and 28 days. Finally, Figure 2 shows the patient numbers per cohort by day, illustrating the relatively low patient numbers from Day 14 onward.

**Figure 3:**
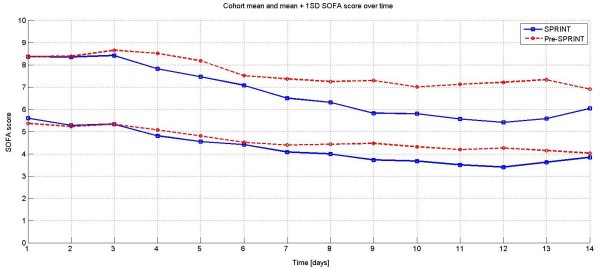
**Mean and Mean +1 SD lines for total SOFA score for the first 14 days for both cohorts**. By Days 3 and 4 there is a clear separation particularly for the mean + 1 SD values (*P *< 0.05).

Figure 3 shows the mean and mean plus one standard deviation of SOFA score for both cohorts over the first 14 days. It is clear that there is divergence starting at Day 2. In particular, the mean plus one standard deviation line diverges to an increasingly lower value for the SPRINT cohort. This result may explain some of the clear divergence seen as early as two to four days in Figure 1.

**Figure 4:**
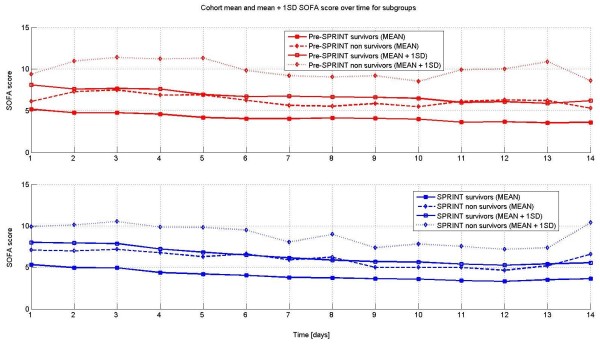
**Mean and Mean + 1SD daily trend lines for survivors and non-survivors for both cohorts**. Pre-SPRINT (top) and SPRINT (bottom).

Figure 4 shows the daily trend of mean and mean plus one standard deviation of the total SOFA score for both cohorts split between survivors and non-survivors. As expected, survivors had lower SOFA scores throughout the time period (*P *< 0.01), and were similar or lower for SPRINT (*P *< 0.01).

The distributions and trends by day for the individual SOFA score components are shown in Appendix B in Additional File 2. However, there were no visible or clinically significant differences between the two cohorts in the distributions for each component. SPRINT patients did tend to have slightly lower median values or IQR, where different, one to two days earlier than Pre-SPRINT patients in some cases.

Examining organ-failure-free days (OFFD), SPRINT OFFD = 1,396 out of 3,356 total possible days (41.6%) were higher than Pre-SPRINT OFFD = 1,172 out of 3,211 (36.5%), which are significantly different (*P *< 0.0001). For individual organ (component) failures (IOF), SPRINT = 2,681 of (Max 5 × 3,356 total possible) or 16.0%, which was lower than Pre-SPRINT = 3,049 out of (5 × 3,211 total possible) or 19.0%, with (*P *< 0.0001). These results indicate that organ failures were reduced in both numbers and time over which failures were experienced with SPRINT. This reduction should have an impact on mortality given the close correlation between organ failure, SOFA score metrics and mortality in several studies.

**Figure 5:**
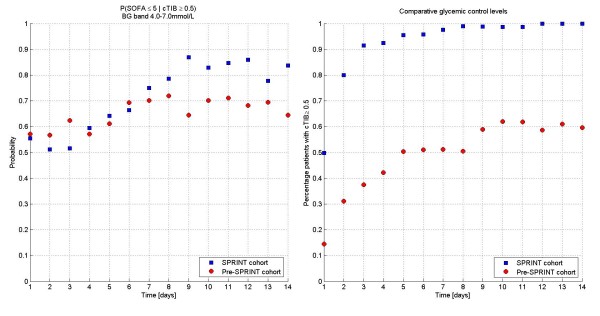
**Conditional probability analysis**. Conditional probability of SOFA ≤5 given cTIB ≥0.5 (A) is equivalent for both cohorts, as expected, while the cohorts differ in the percentage of patients achieving cTIB ≥0.5 (B).

Figure 5 shows the conditional probability (P(SOFA ≤5 | cTIB ≥0.5)) of SOFA ≤5 given cTIB ≥0.5 for each day with the percent of patients achieving cTIB ≥0.5. The conditional probabilities are not statistically significantly different until Day 14. Through Day 8 they are effectively equivalent, which should be expected if good control yields faster reduction of SOFA score, as this physiological and clinical outcome should be independent of the manner in which TGC is delivered. Differences after Day 8 could be due to several factors, including different patient management to less acute wards, or differences (not statistically significant in Table 1) between cohorts, as well as evolution of different treatment regimes such as mechanical ventilation or steroid use. It is also clear (right panel) that far more patients received and maintained good control under SPRINT providing some of the difference in Figure 1.

**Figure 6:**
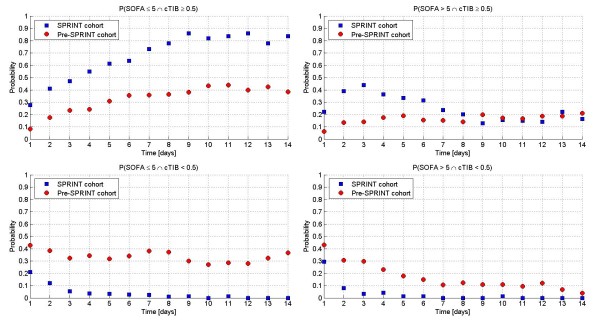
**Joint probabilities for all four combinations of SOFA score and cTIB, for both cohorts**. Joint probability analysis of SOFA score and cTIB for all four combinations given a SOFA threshold of 5 and a cTIB threshold of 0.5.

Figure 6 shows the four joint probability cases. It is clear from the Figure that: (1) SPRINT patients had a higher joint probability of SOFA ≤5 with good control as seen in Panel A, which is essentially the lines in Figure 5 (left) scaled by the lines in Figure 5 (right); (2) Panel B shows those patients who do not improve in SOFA score despite receiving good control, and are effectively equivalent after six to eight days for both cohorts, indicating those patients who simply do not recover regardless; (3) The lines in Figure 1 are the sum of Panels A and C, where, for the retrospective cohort, Panel C shows that many patients can have SOFA ≤5 despite poor control, as might be expected clinically; (4) The remainder in Figure 1 from the curves up to 100% (going up) are thus the sum of Panels B and D; (5) SPRINT patients had effectively no patients in panels C and D for poor control, per Figure 5 (right panel), after three days; (6) The Pre-SPRINT patients (no SPRINT patients) in Panel D are thus those who, if they had received good control, would have moved to either Panel A or B. There are enough patients in Panel D to cover the gap between the cohorts in Figure 1.

These conditional and joint probabilities indicate that while good control is not a requirement for SOFA ≤5, it is not harmful and, further, does provide a greater likelihood of reaching SOFA ≤5 for approximately 10 to 15% of patients.

**Figure 7:**
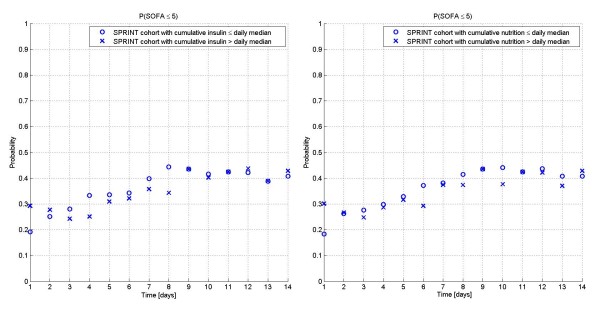
**Impact of insulin and nutrition on SOFA scores in SPRINT**. Comparison of Insulin (A) and nutrition (B) cumulative rates for SPRINT patients with SOFA ≤5, broken into those with greater than the cumulative daily median value for the cohort, and those with less. The results indicate that SPRINT patients with SOFA ≤5 were equally likely to receive greater or less insulin and/or nutrition than the entire cohort (all SOFA scores).

To ensure the results in Figure 5 are not due to giving more or less insulin or nutrition compared to the rest of the SPRINT cohort, Figure 7 shows the percent of patients each day with SOFA ≤5 who received more or less than the cumulative median insulin or nutrition rate for the whole cohort up to that day. It is clear that there are no significant differences (*P *= 0.28 for insulin and *P *= 0.13 for nutrition) in these interventions for SOFA ≤5 patients versus the whole cohort (all SOFA values). Hence, SOFA ≤5 results were not obviously linked to receiving different insulin or nutrition than the entire cohort.

## Discussion

Only Vincent *et al*. [5] have examined daily SOFA score trajectories showing its ability to capture morbidity and mortality over time. To the authors' knowledge, this paper presents the first evaluation of the impact of a clinical intervention using SOFA score and its change over time.

The main results in Figure 1 clearly show that organ failure resolved faster with effective TGC under the SPRINT protocol than for a retrospective control, given similar initial and maximum SOFA scores. While the results show a consistent reduction in SOFA score and organ failure for all patients, this reduction is more evident for higher percentile, more critically ill patients (mean + 1SD, 83^rd ^percentile) with higher SOFA scores.

Figures 5 and 6 use conditional and joint probabilities to relate TGC performance and SOFA score outcomes. Figure 5 clearly shows that effective TGC and SOFA ≤5 are related for at least the first eight days and are not statistically different (*P *> 0.06) until Day 14. This equivalency reflects the hypothesis of low SOFA score being related to effective TGC and should not depend on how that TGC was delivered. Hence, it is primarily the difference in the percent of patients receiving effective TGC that separates these cohorts.

Finally, Figure 6 delineates the different combinations of TGC effectiveness and SOFA outcome. As might be expected, Panels B and C show that some patients never obtain SOFA ≤5 with good control, regardless of cohort, while others achieve SOFA ≤5 despite poorer control (cTIB < 0.5). Thus, it is panel D that indicates, in this context, that TGC (under SPRINT) might have its greatest benefit on the 10 to 15% of patients for whom improved control would not be harmful and may well define the difference in the curves of Figure 1 separating the cohort.

There is no further specificity to the results in terms of which specific patients or sub-groups may have driven this difference. SPRINT reported no statistically significant difference (*P *> 0.35) between survivors and non-survivors for any glycemic outcome, diabetic status, diagnostic code, insulin infused or carbohydrate nutrition, and the resultant mortality [21]. In contrast, the retrospective cohort maintained statistically significant associations for all glycemic outcomes except average blood glucose and insulin infused. These results imply, as above, that glycemic outcome was the main difference in these two cohorts and their outcomes.

Further small differences in Figure 5 after eight days reduce the link between effective TGC of any sort and lower SOFA score. These may have several causes, but it should also be noted that there is a relatively large mortality difference in patients with greater than five-day stay in ICU between these cohorts. Other differences in cohort, patient management or unreported changes in care may also play a role. Figure 2 reflects some of these issues as the Pre-SPRINT cohort undergoes far faster changes in numbers than SPRINT over Days 4 to 10, crossing at Day 8.

Physiologically, hyperglycemia can have lasting cellular level impact, even during subsequent euglycemia, due to over production of superoxides [15,17], leading to further damage and complications. Similarly, exposure to elevated blood glucose levels over 7.0 mmol/L resulted in significant 33 to 66% reductions in immune response effectiveness [22,24], thus increasing the risk of further infection and complications. These points indicate that it is the long-term, cumulative quality of control that may be critical, and SPRINT provided tighter, less variable and more consistent TGC than the Pre-SPRINT cohort.

This study used cTIB ≥0.5 as a daily metric to assess the consistency of tight control. This value also clearly discriminated the SPRINT (92% of cohort met this target at three days) and Pre-SPRINT (37%) cohorts, clearly showing the difference in quality of control despite similar cohort median values (6.0 mmol/L SPRINT vs 7.2 mmol/L Retrospective). Clinically, this metric sets a potential benchmark for assessing glycemic performance that is directly associated, in this study, with a clinical outcome.

With respect to limitations, a threshold of SOFA ≤5 was chosen to represent a relatively well patient expected to survive. However, there are no clearly defined standards for this choice, but the literature shows that this approach is conservative. Low numbers for observing this phenomenon may also be a limitation, particularly after 14 days, where Figure 2 shows only 75 and 67 patients remaining in each cohort. Note that Christchurch Hospital does not have a high dependency or "step down" unit, which could affect any comparison of these patient numbers or results to some other units.

Further, potential confounders exist in any retrospective analysis as therapy approaches evolve over time. In this case, there were no specifically implemented changes in mechanical ventilation therapy, steroid use, or specific sepsis campaigns. However, clinical practice is always evolving and staff turnover has an impact as well. Hence, these results must await repetition in a randomised setting. That said, the impact of SPRINT on nutritional inputs and carbohydrate loading is a significant clinical difference and practice change outside the resulting glycemic control, although it did not have a notable impact in Figure 7 within the cohort. Overall, the results presented, despite potential limitations, should justify a randomised trial to test this approach.

It should also be noted that both the OFFD and IOF results supported the overall result that organ failure was reduced under SPRINT in both number and the time experienced. However, it should be noted that IOF could be lower if early mortality is higher as there is less time to develop organ failures before death. However, both cohorts reached similar maximum SOFA scores in similar times. In addition, the equivalent lengths of stay, combined with greater OFFD with SPRINT TGC indicates that this case has not occurred.

Finally, SPRINT showed a significant improvement in mortality for those patients staying five days or longer in ICU, so analysing this group separately might be interesting. Repeating the analysis of Figure 1 for both cohorts split into those staying five plus days and those staying less than five days had two main results. Those staying less than five days (median two days) had lower SOFA scores and thus significantly higher percentages of patients (approximately 25% absolute) with SOFA ≤5 for Days 1 to 4 compared to those who stayed five days or longer. Thus, those staying longer had higher SOFA scores at admission and maximum (one day), and represented a more critically ill cohort. These longer stay patients make up the entire curve of Figure 1 from Day 5 onward. Thus, the results are effectively unchanged from what is presented here if this division of the cohorts is considered.

## Conclusions

This study presents results from a unique analysis that evaluates the impact of an intervention in terms of daily organ failure status. Three main conclusions are drawn from this analysis.

First, TGC using SPRINT had a significant effect in resolving organ failure both faster and for a greater percentage of the cohort compared to a matched retrospective cohort. These results were independent of the initial, maximum and component organ failure scores, and independent of the time to reach the similar maximum SOFA score value, indicating the result is spread across several factors. It also decreased total organ failure days and increased organ failure free days.

Second, the differences in SOFA score seen here can be related to the tightness and consistency of TGC provided, as assessed by a cumulative time in band metric. The cTIB metric and the threshold used provide an initial benchmark result linking the quality of control to a clinical outcome.

Third, The use of daily organ failure status and specifically of the percentage of patients with resolved organ failure provides a unique means of assessing the impact of this (or any similar) intervention. The differences observed reflect differences in morbidity for which the SOFA score was designed. As such they also reflected the mortality differences observed in these cohorts in the original study, and did so at the same ICU length of stay where changes in hospital and ICU mortality were observed in the original study. Thus, the total SOFA score used on a daily basis can provide significant insight into the progress and efficacy of an intervention.

All of these main conclusions remain to be prospectively tested. However, this analysis highlights several key outcomes with respect to the impact of TGC and its assessment using the SOFA score, as well as providing some insight into potentially improved methods of assessing similar future randomised intervention studies.

## Key messages

• Effective, tight glycaemic control under the SPRINT protocol to a mean of 6.0 mmol/L mitigated organ failure faster than conventional, less tight control at a higher mean level of 7.2 mmol/L

• Tight glycaemic control in this study reduced total organ failures and increased organ failure free days, and was linked to improved SOFA score outcomes

• Tight glycaemic control had no impact on the maximum SOFA scores or the day on which they occurred indicating that its affect on organ failure occurs after the first one to two days

• Daily SOFA scores provide a significant indicator of the impact of glycaemic control on patient morbidity and mortality

• The reduction in organ failure as measured by the SOFA score is hypothesised as the causative factor of the reduced mortality in the SPRINT cohort for patients who stayed in the ICU three days or longer

## Abbreviations

APACHE: Acute Physiology and Chronic Health Evaluation; cTIB: cumulative Time in Band; ICU: intensive care unit; IOF: individual organ failures; OFFD: organ failure free days; SOFA: Sequential Organ Failure Assessment; SPRINT: Specialised Relative Insulin and Nutrition Titration; TGC: tight glycaemic control

## Competing interests

The authors declare that they have no competing interests.

## Authors' contributions

JGC, GS, ALC and JL conceived and developed the SPRINT protocol. GS implemented the protocol with staff at Christchurch Hospital. LP, CGP, DH, ALC, JGC, GS, TD, J-CP, JL, and KTM assisted in data collection and/or the analysis and interpretation of the data and/or statistical analysis. JGC, J-CP, ALC and KTM drafted the manuscript primarily although all authors made contributions. All authors approved the final manuscript.

## Supplementary Material

Additional file 1 **SPRINT Protocol details and differences to other TGC protocols**. A more detailed description of SPRINT and specific, unique differences to other protocols. Click here for file

Additional file 2 **Supplementary data on component SOFA scores**. Distributions and trends by day for the individual SOFA score components Click here for file
