# Supplementary material for: Organ failure and tight glycemic control in the SPRINT study
Source: Crit Care. 2010 Aug 12;14(4):R154. doi: 10.1186/cc9224 (PMC2945138; doi:10.1186/cc9224)
Supplement: Additional file 1 — SPRINT Protocol details and differences to other TGC protocols. A more detailed description of SPRINT and specific, unique differences to other protocols. [file cc9224-S1.DOCX]

**SPRINT protocol details and differences to other TGC protocols**

**A-1: The Protocol**: The entry criterion for the SPRINT protocol was a blood glucose measurement greater than 8 mmol/L on two occasions during standard patient monitoring. Patients are sometimes put on SPRINT at the discretion of the clinician if the blood glucose levels were consistently greater than 7 mmol/L in severe critical illness.

Hourly blood glucose measurements are used to ensure tight control [28]. Two-hourly measurements are used when the patient is stable, defined as 3 consecutive 1-hourly measurements in the 4.0-6.0 mmol/L band [28, 29], or when an arterial line is not present. SPRINT is stopped when the patient is adequately self regulating, defined as 6 or more hours (three 2-hourly measurements) in the 4.0-6.0 mmol/L band with over 80% of goal feed rate and a maximum of 2U/hour of insulin [28, 29]. Finally, all measurements are made with bedside glucometers with standard error of 7-12%.

Total insulin prescribed by SPRINT is limited to 6U/hr to minimise saturation and the administration of ineffective insulin [31, 53-55]. Insulin is given predominantly in bolus form for safety, avoiding infusions being left on at levels inappropriate for evolving patient condition. Occasionally, doctors prescribed a background insulin infusion rate of 0.5–1.0 U/hr, primarily for patients known to have Type II diabetes, and the insulin bolus recommendations from SPRINT are added to this rate. A background rate of 0.5–1.0 U/hr can also be mandated in patients with Type I diabetes.

Goal enteral nutrition rates are approximately 25 kcal/kg/day of RESOURCE Diabetic (Novartis Medical Nutrition, Minneapolis, MN) or Glucerna (Abbott Laboratories, Chicago, IL) with 34-36% of calories from carbohydrates. Maximum and minimum nutrition rates are 7.5 to 25 kcal/kg/day with 2.7 to 9 kcal/kg/day from carbohydrate. Thus, an 80kg male would receive maximum of 2000 kcal/day and a minimum of 600 kcal/day, with 216-640 kcal/day from carbohydrate, exceeding the minimum level below which there is an increased risk of bloodstream infections [38]. These guidelines are detailed in [56] and are effectively equivalent to the ACCP/SCCM guidelines [36].

Figures A.1-A.4 show the SPRINT wheels that define the protocol, as well as stopping and measurement frequency criteria flowcharts. These wheels are used each 1-2 hours to determine the insulin and nutrition interventions. As noted, they effectively titrate interventions based on response to both inputs, effectively titrating on patient-specific insulin sensitivity displayed by that response.

**A-2: Specific Features and Differences of SPRINT**: The SPRINT approach to TGC was unique in several areas from almost all other protocols. Specific differences are outlined below in terms of broad areas wherein differences lie:

- **Nutrition and Carbohydrate Intake**:
  - SPRINT directly controlled both insulin and nutrition where virtually all other protocols do not explicitly account for nutrition or carbohydrate intake. Hence, they dose insulin “blind” to carbohydrate intake
  - SPRINT specified the specific enteral and other nutritional sources, using low carbohydrate formulations with only 35-40% calories from carbohydrate. As a result, SPRINT had lower carbohydrate loading but within the guidelines for improved mortality reported by [37].
- **Interventions and TGC Approach**: The following issues defined the salient differences between SPRINT and other protocols in terms of how SPRINT is implemented, its limits on interventions, and how it titrates interventions on patient-specific insulin sensitivity (1/insulin resistance):
  - SPRINT controls both insulin and nutrition in concert and within safe ranges.
  - SPRINT specified insulin and nutrition interventions based on patient-specific insulin sensitivity. Thus, these interventions are not based on glycemic response, as in almost all other algorithms, but on glycemic response to patient-specific interventions.
    - Insulin sensitivity is a direct, patient-specific response to the inflammatory and counter-regulated critically ill patient state. Hence, it makes sense to titrate on estimates of this value to adjust interventions.
    - This approach cannot be taken without direct knowledge of the carbohydrate dosing given, making SPRINT unique.
  - SPRINT had a relatively quite low maximum insulin limit of 6U/hour to avoid saturation effects and also the risk of hypoglycemia. Most published protocols utilise much higher maximum rates of insulin dosing, with some allowing up to 10-30 units or more per hour (e.g. [57, 58]).
    - Uniquely, SPRINT gives insulin in bolus form for safety as an infusion can be left running when not desirable and a bolus cannot.
  - SPRINT stops insulin if a blood glucose drop is “large” defined as 1.5 mmol/L in 1-2 hours with a current BG measurement less than 7 mmol/L (see Figure A.2), which is above the target band of 4.0-6.0 mmol/L. In contrast, many published protocols do not stop insulin unless hypoglycemia occurs and/or reduce the rate when insulin had passed below the glycemic target band [27, 58], at which point it is often too late to avoid severe hypoglycemic events.
  - SPRINT modulates its interventions very slowly with over 90% of possible outcomes requiring a change of only 1U/hour of insulin and/or 10% change in feed rate. This relatively slow approach to titrating blood glucose minimised rapid changes and the potential for hypoglycemia.
  - SPRINT measured and intervened 1-2 hourly with no 3-4 hourly or longer intervals, ensuring tighter potential control over highly dynamic patients compared to most protocols that allow 4-hourly measurement, which for highly dynamic patients can result in loss of control as patient condition evolves.
  - SPRINT measured 2-hourly only in stable patients. Stable patients were defined as in the target band of 4-6 mmol/L and had relatively high insulin sensitivity (3U/hour or less of insulin and 60% or more of patient-specific goal feed rate).
    - Higher insulin sensitivity and correspondingly lower insulin usage implies a patient in relatively better condition and one with less change in glycemia due to dynamic changes in patient condition.
    - This is a unique definition of stability compared to other protocols, and a more rigorous or harder definition to meet than merely being well controlled or in a target band.

Overall, SPRINT focused on patient-specific response to insulin and nutrition interventions, or effective insulin sensitivity to titrate interventions and define stability. As a result, with more frequent measurement, it was able to obtain tighter control across the range of dynamics and variability seen in these patients.

As a result, the following glycemic outcomes were observed that are relevant to the tightness of control and/or unique to this protocol:

- SPRINT had higher times in all glycemic bands than other protocols [41] and thus tighter (less variable) control. In particular, the 97^th^ percentile patient over 50% of all blood glucose values in a 4.0-7.0 mmol/L range, which is tighter than other reports.
- SPRINT had very low hypoglycemia (2% by patient) than other protocols with similar targets and, equally uniquely, TGC reduced hypoglycemia by 50% from the retrospective comparator cohort.
- SPRINT provided more consistent control on a per-patient basis, in both tightness of control (variability) and target value (median glycemia) than all other protocols who reported per-patient values. The middle 50% of SPRINT patients had median blood glucose within a 1.1 mmol/L wide band (5.5-6.6 mmol/L). Many TGC protocols do not report per patient results for both median and variability so comparison is difficult, but spread of reported patient means has been much larger, as well as a significant discussion point for several trials [25, 41, 59].
  - Figure A.5 shows the median and IQR per-patient blood glucose distributions as cumulative distribution functions for both cohorts.
  - Figure A.6 shows the cohort to cohort comparison of blood glucose
- SPRINT gave more insulin on a cohort and per-patient basis, but had less hypoglycemia, contrary to many published studies and analyses [60].
- SPRINT gave less carbohydrate and total nutrition on average across the cohort, but the median SPRINT patient received more nutritional inputs (although lower carbohydrate loading) than in the Pre-SPRINT cohort (see Table 2 in [21]).
- Within this SPRINT cohort, there was no statistical link (P>0.4) between glycemic outcome (average, range, maximum) and outcome. No other study reported this decoupling of outcome glycemia and mortality.

Hence, SPRINT provided very tight control on a cohort and per-patient basis compared to all other published results (e.g. [33]), and succeeded in decoupling mortality and glycemic outcome within the SPRINT cohort, which is also unique. High times in band a low variability are key aspects in reducing the well reported physiological impact of hyperglycemia on inflammation and counter regulation and immune response, and thus, as hypothesised in this paper, on organ failure.


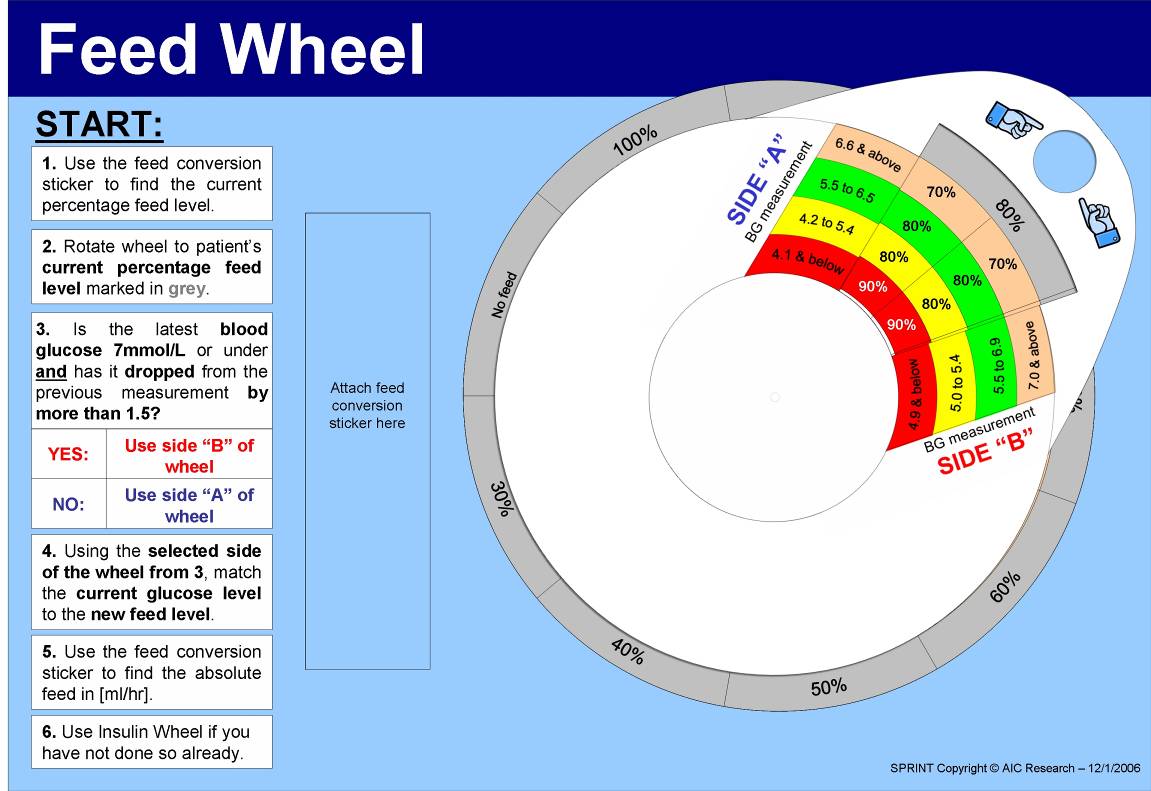


(a)


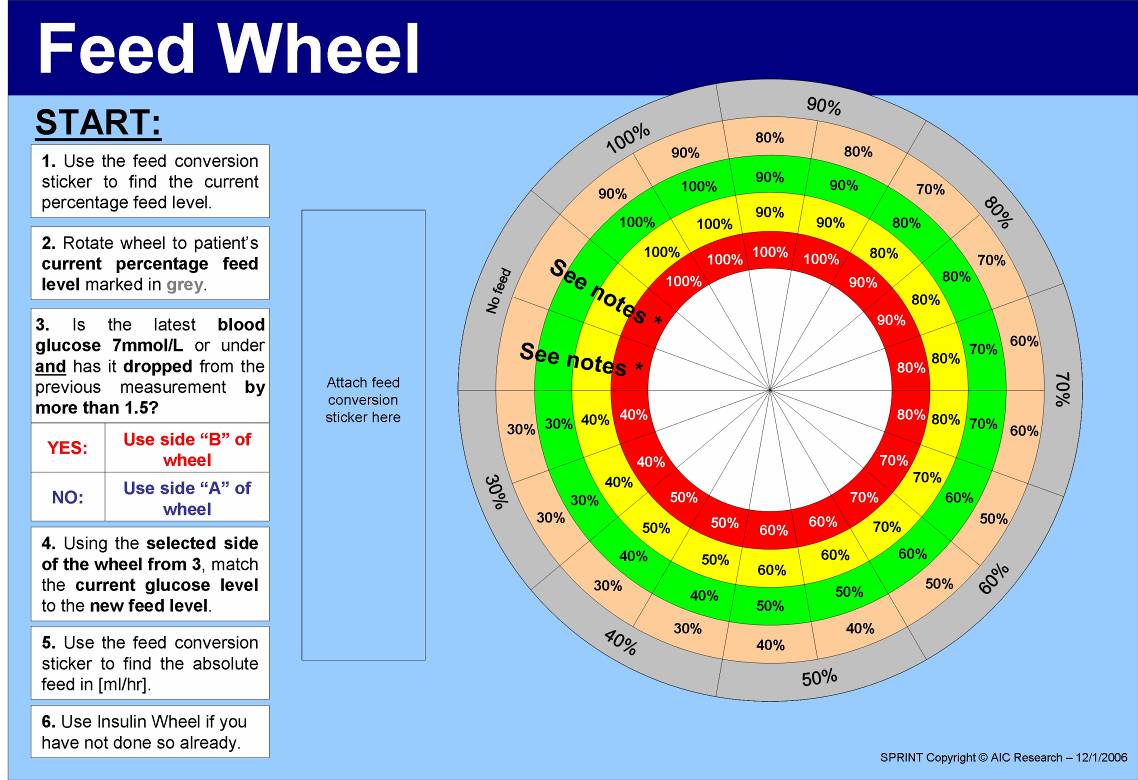


(b)

**Figure A.1:** The SPRINT feed wheel with dial (a) and with dial removed (b). (Blood glucose values are in mmol/L, to convert to mg/dL multiply by 18).


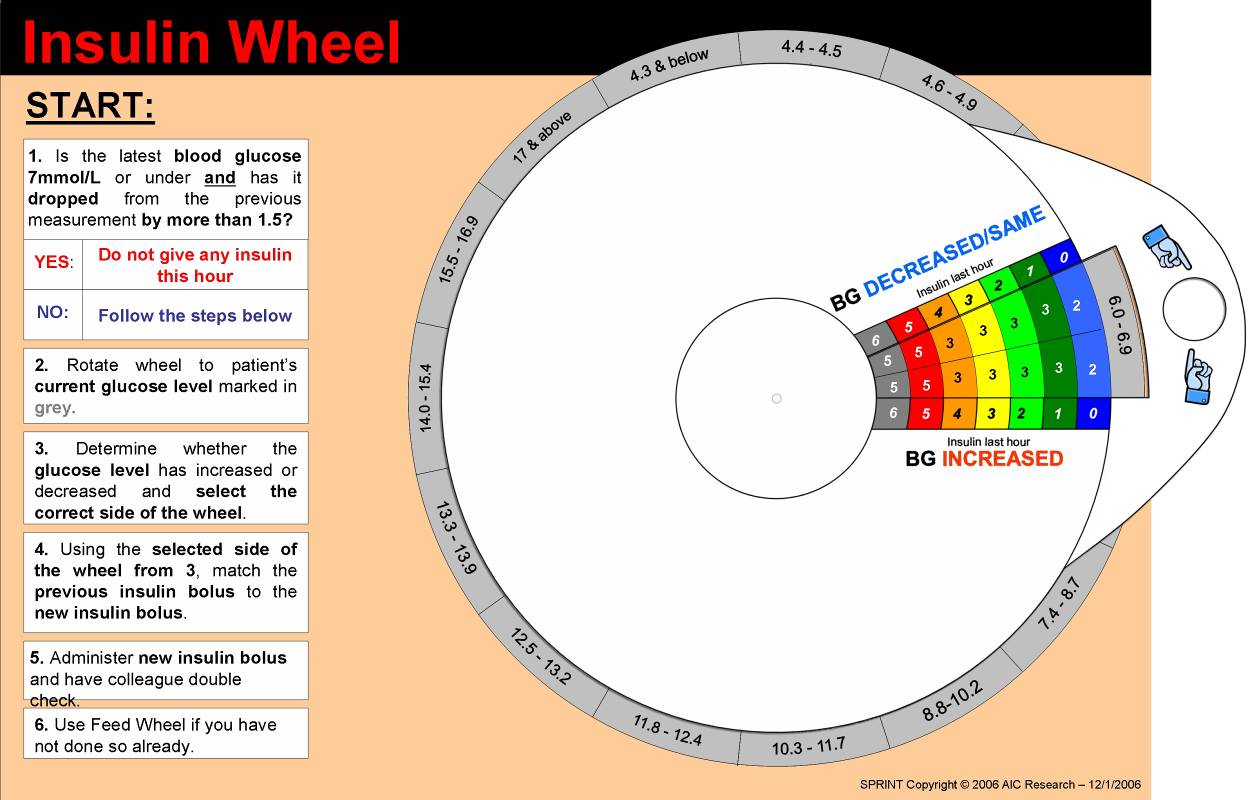


(a)


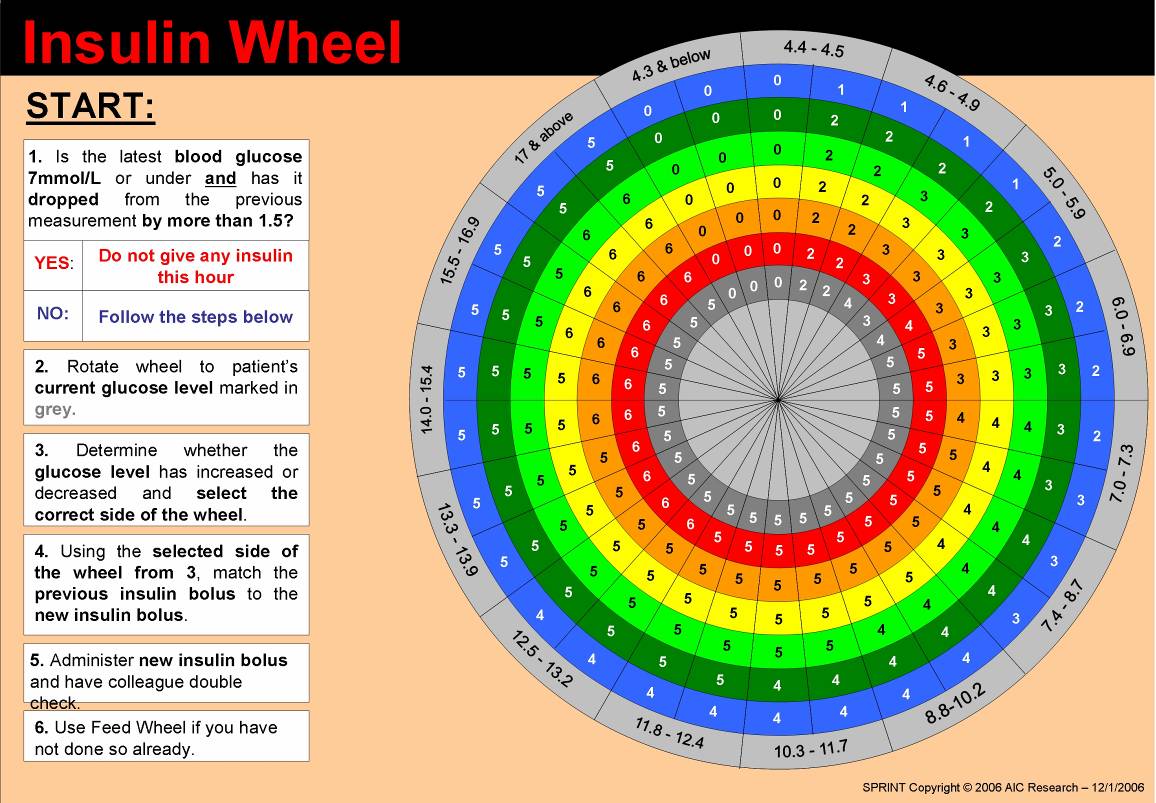


(b)

**Figure A.2:** The SPRINT Insulin wheel with dial (a) and with dial removed (b). (Blood glucose values are in mmol/L, to convert to mg/dL multiply by 18).

**Figure A.3:** Flow chart specifying guidelines for measuring blood glucose level two-hourly.

**Figure A.4:** Flow chart specifying guidelines for stopping the SPRINT protocol


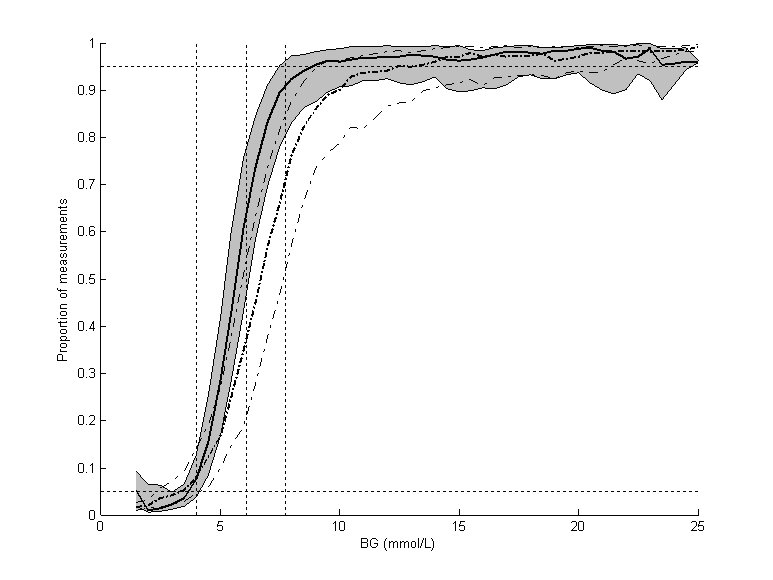


Figure A.5: Per-patient cumulative distribution of blood glucose results for SPRINT and Pre-SPRINT

Figure A.6: Cohort wide cumulative distribution of blood glucose values for SPRINT and Pre-SPRINT cohorts.
