# Supplementary material for: Organ failure and tight glycemic control in the SPRINT study
Source: Crit Care. 2010 Aug 12;14(4):R154. doi: 10.1186/cc9224 (PMC2945138; doi:10.1186/cc9224)
Supplement: Additional file 2 — Supplementary data on component SOFA scores. Distributions and trends by day for the individual SOFA score components [file cc9224-S2.DOCX]

**Supplementary data on component SOFA scores**

Figures B.1 show the component SOFA scores over time in box and whisker format (median, IQR and 90% CI) including outliers. The main points to note are that there is little clinical difference between Pre-SPRINT and SPRINT on a cohort basis for most days or components. Particular differences or points of note in Figures B.1 (in the order A-E shown in the figure) including p-values where daily distributions are significantly different, are summarised:

- **Respiratory function**: Respiratory failure is common and the plots are little different across the days between cohorts. The SPRINT cohort shows a wider range and less failure in this component score in days 2, 4 and 5 and in the last 3 days. Day 15 has significantly different distributions between groups, p = 0.032. All other days have p > 0.05.
- **Cardiovascular function**: Cardiovascular function scores are common and differences in median and/or IQR are evident on days 2-5 with SPRINT having lower scores than the Pre-SPRINT cohort. All days have p > 0.05 in comparing the two groups.
- **Renal function**: there is very little renal failure in either cohort with the box and whiskers (0 – 95%) on the SOFA = 0 line. Outliers are shown as the red crosses. Day 9 has significantly different distributions between groups, p = 0.049. All other days have p > 0.05.
- **Liver function**: there is very little liver failure in either cohort with the box and whiskers (0 – 95%) on the SOFA = 0 line. Outliers are shown as the red crosses. Day 3 has significantly different distributions between groups, p = 0.026. All other days have p > 0.05.
- **Coagulation**: This score is similar between cohorts, with the only significant difference seen on day 7 where SPRINT median and IQR and 90% CI are all 0 and are non-zero for the SPRINT cohort. Days 8, 9 and 11-20 have significantly different distributions between groups, p < 0.020 in all cases. All other days have p > 0.05.

The overall result is that individual SOFA score components show no significant trends favouring one or another component.


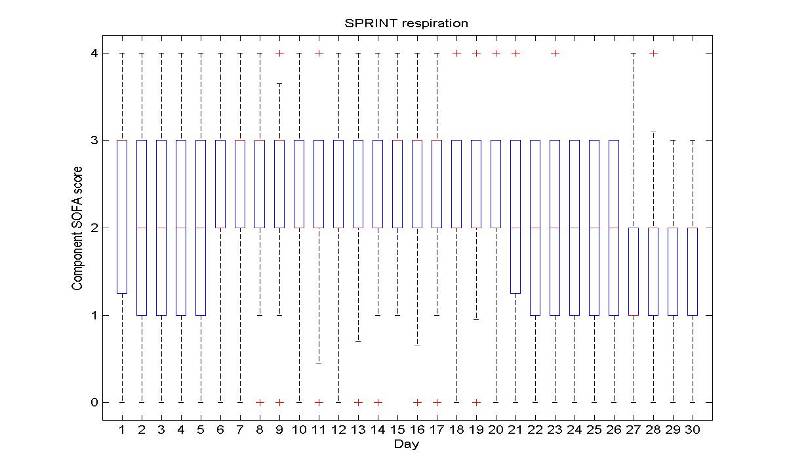

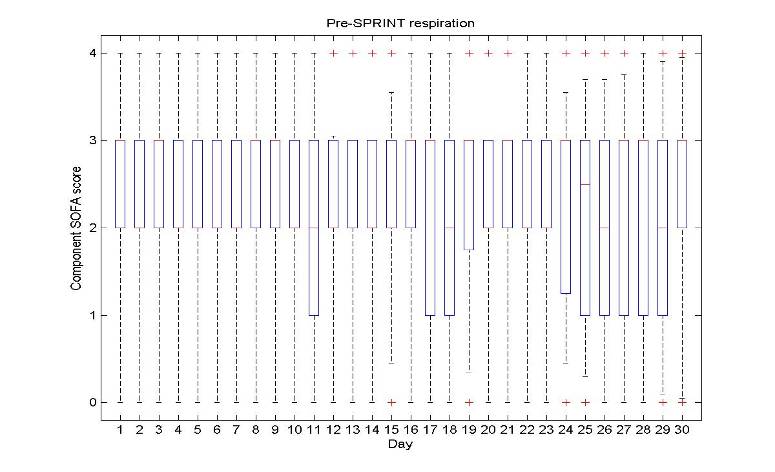


**Pre-SPRINT SPRINT**

**A. Respiratory function score 0 – 4 for days 1 - 30**

**B. Cardiovascular function score 0 – 4 for days 1 - 30**


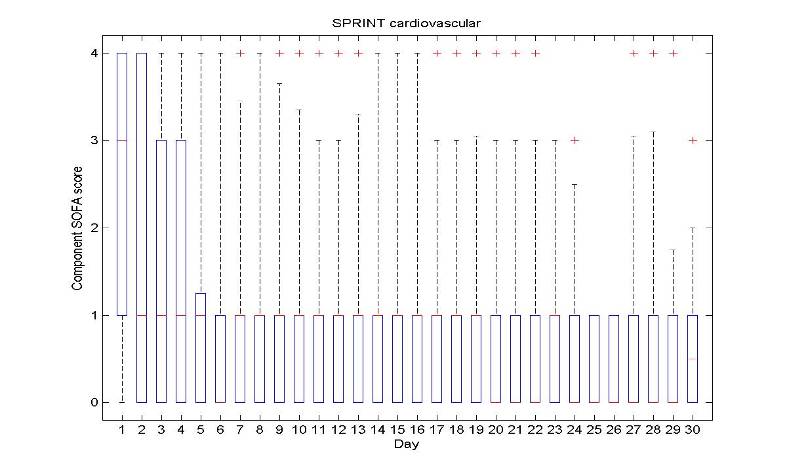

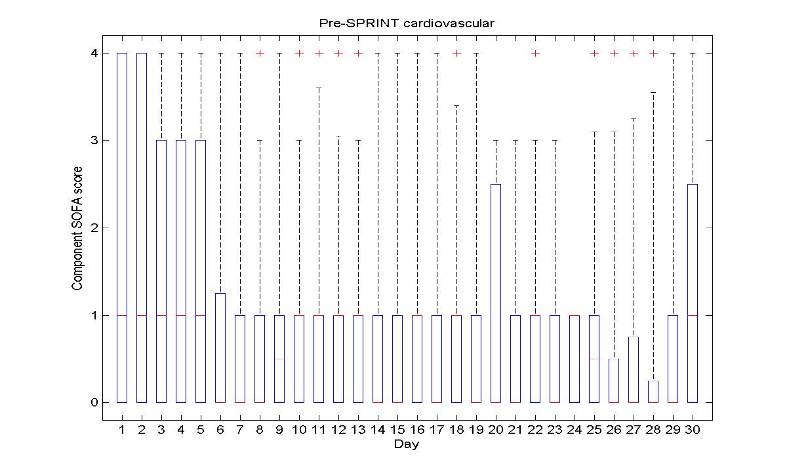


**C. Renal function score 0 – 4 for days 1 - 30**


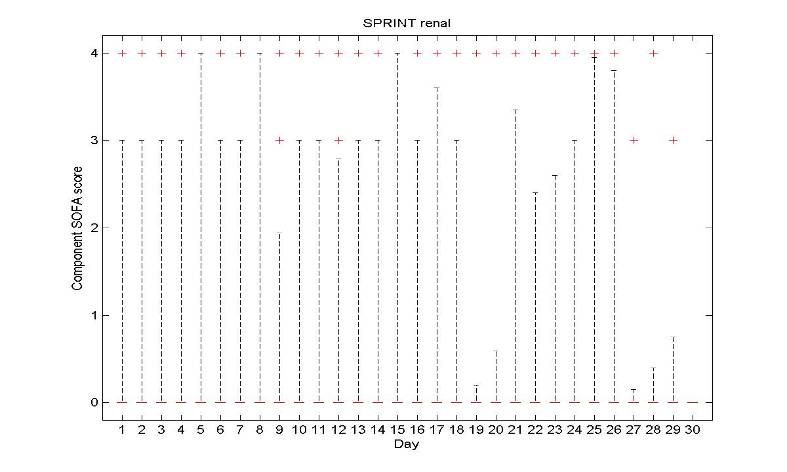

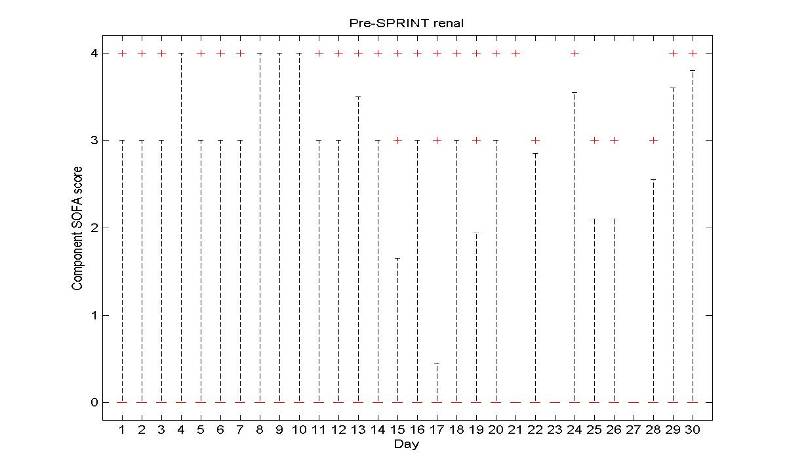


**Figure B.1**: SOFA score box and whisker plots for SOFA score components for the Pre-SPRINT cohort (left column) and SPRINT (right column) over time for: **A**) Respiratory function; **B**) Cardiovascular function; and **C**) Renal function.

**D. Liver function score 0 – 4 for days 1 - 30**


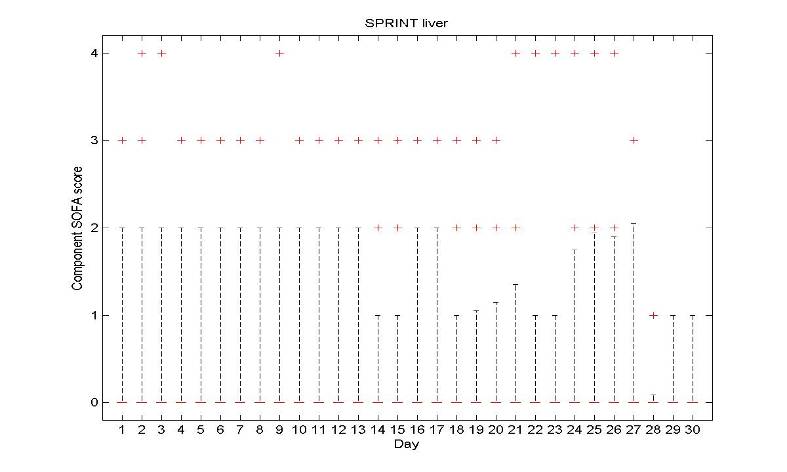

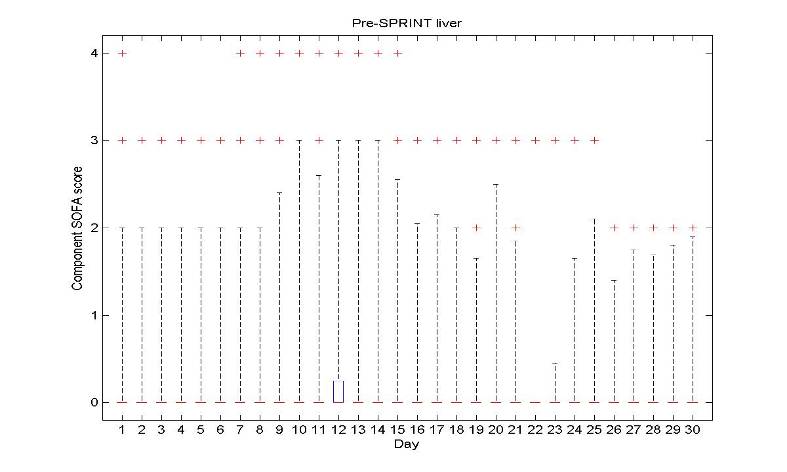


**E. Coagulation function score 0 – 4 for days 1 - 30**


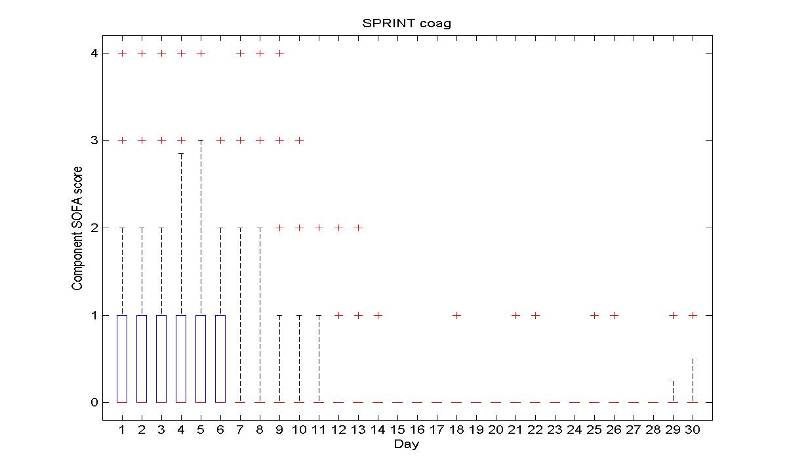

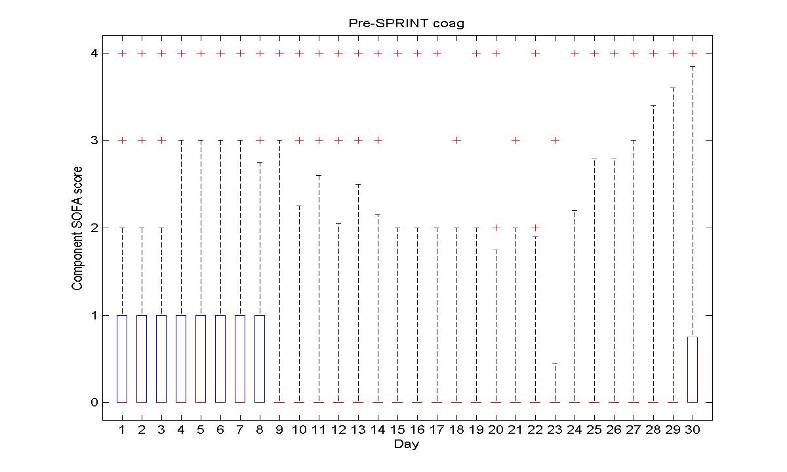


**Figure B.1 (continued)**: SOFA score box and whisker plots for SOFA score components for the Pre-SPRINT cohort (left column) and SPRINT (right column) over time for: **D**) Liver function; and **E**) Coagulation function..
